# Changes in the Extracellular Matrix in Endometrial and Cervical Cancer: A Systematic Review

**DOI:** 10.3390/ijms24065463

**Published:** 2023-03-13

**Authors:** Tjaša Padežnik, Anja Oleksy, Andrej Cokan, Iztok Takač, Monika Sobočan

**Affiliations:** 1 Department of Gynaecology and Obstetrics, Faculty of Medicine, University of Maribor, Taborska Ulica 8, 2000 Maribor, Slovenia; 2 Divison for Gynaecology and Perinatology, University Medical Centre Maribor, Ljubljanska Ulica 5, 2000 Maribor, Slovenia; 3 Department for Pharmacology, Faculty of Medicine, University of Maribor, Taborska Ulica 8, 2000 Maribor, Slovenia

**Keywords:** extracellular matrix, endometrial cancer, cervical cancer

## Abstract

Endometrial and cervical cancers are the two most common gynaecological malignancies and among the leading causes of death worldwide. The extracellular matrix (ECM) is an important component of the cellular microenvironment and plays an important role in developing and regulating normal tissues and homeostasis. The pathological dynamics of the ECM contribute to several different processes such as endometriosis, infertility, cancer, and metastasis. Identifying changes in components of ECM is crucial for understanding the mechanisms of cancer development and its progression. We performed a systematic analysis of publications on the topic of changes in the extracellular matrix in cervical and endometrial cancer. The findings of this systematic review show that matrix metalloproteinases (MMP) play an important role impacting tumour growth in both types of cancer. MMPs degrade various specific substrates (collagen, elastin, fibronectin, aggrecan, fibulin, laminin, tenascin, vitronectin, versican, nidogen) and play a crucial role in the basal membrane degradation and ECM components. Similar types of MMPs were found to be increased in both cancers, namely, MMP-1, MMP-2, MMP-9, and MMP-11. Elevated concentrations of MMP-2 and MMP-9 were correlated with the FIGO stage and are associated with poor prognosis in endometrial cancer, whereas in cervical cancer, elevated concentrations of MMP-9 have been associated with a better outcome. Elevated ADAMTS levels were found in cervical cancer tissues. Elevated disintegrin and metalloproteinase with thrombospondin motifs (ADAMTS) levels were also found in endometrial cancer, but their role is still unclear. Following these findings, this review reports on tissue inhibitors of ECM enzymes, MMPs, and ADAMTS. The present review demonstrates changes in the extracellular matrix in cervical and endometrial cancers and compared their effect on cancer development, progression, and patient prognosis.

## 1. Introduction

Endometrial cancer (EC) is one of the most common malignancies of the female genital system and the sixth most common type of all cancers worldwide [1,2,3]. The incidence is higher in developed countries, presumably due to the higher prevalence of obesity and an increase in life expectancies [4]. Based on clinicopathological and molecular genetic features, it can be classified into two main major types: type I and type II. Type I (endometrioid) EC is found in approximately 80% of cancer patients, is oestrogen-dependent, and develops from atypical hyperplasia. It usually occurs in younger, pre-menopausal women and has a good prognosis with a five-year survival rate of 80%. In contrast, type II (non-endometrioid) EC is oestrogen independent and develops from atrophic endometrium or endometrial polyps. It occurs usually in older, post-menopausal women and has a poor prognosis with a five-year survival rate of only 17% [4,5]. Patients with type I endometrial cancer are usually diagnosed at an early stage (International Federation of Gynecology and Obstetrics (FIGO) stages I and II), whereas type II endometrial cancer is highly aggressive and therefore also found many times in later stages (FIGO stages III and IV) [6]. The most common treatment of endometrial cancer is surgery followed by systemic therapy and/or radiotherapy in higher risk EC [6,7].

Cervical cancer (CC) is also one of the most common gynaecological cancers worldwide [8,9]. The leading cause of invasive cervical cancer is persistent infection with high-risk human papillomaviruses (HPVs) [9,10,11]. Oncogenic HPV types 16 and 18 have the highest prevalence [12]. Two major types of cervical cancer are cervical squamous cell carcinomas and cervical adenocarcinomas [13]. Treatment of cervical cancer depends on the clinical stage of cancer. Patients in the early stages usually undergo surgery or radiotherapy, whereas patients in advanced stages are treated with systemic therapy or other sequencing treatment based on multidisciplinary tumour board decisions. However, cancer recurrence in patients who have advanced-stage cancer is high with a five-year survival rate lower than 50% [14,15,16].

The extracellular matrix (ECM) is an important component of the cellular microenvironment and plays an important role in developing and regulating normal tissues and homeostasis. The extracellular matrix of the female reproductive system undergoes extensive structural remodelling. The pathological dynamics of the ECM contribute to several different processes such as endometriosis, infertility, cancer, and metastasis [17]. Tumour cells are surrounded by the ECM. Degradation of the ECM by various enzymes allows tumour cells to invade the local surroundings and metastasize [18]. Components of the ECM such as different glycoproteins, proteoglycans, and others are important for cellular signal transduction and play an important role in the process of carcinogenesis [1]. 

Identifying changes in components of ECM is crucial for understanding the mechanisms of cancer development and its progression. Main components can be used as potential biomarkers and a thorough investigation of their effect on surrounding tissues could lead to the development of new treatment strategies. This review aimed to identify advances in our understanding of changes in the extracellular matrix in cervical and endometrial cancers, analyse their effect on cancer development and progression and compare them. 

## 2. Materials and Methods

Due to the heterogeneity of the addressed topic and the fragmented literature, a conventional systematic review was not considered appropriate. We searched the online database PubMed (https://pubmed.ncbi.nlm.nih.gov, accessed on 20 October 2022) for manuscripts published from January 2010 until December 2021. Search keywords included “endometrial cancer” or “cervical cancer” and “extracellular matrix”. We reviewed the list of all references for further relevant studies. Publications were limited to the English language. Exclusion criteria included review articles, studies involving patients who underwent treatment other than surgical (radiotherapy, chemotherapy, or drug therapy), and articles for which full text was not available. 

We only used studies that involved patients who were not undergoing any type of treatment other than surgical. Chemo- and (or) radiotherapy, or any type of drug therapy could affect normal extracellular matrix; therefore, we would not be able to distinguish between changes caused by therapy and those caused by cancer.

**Figure 1:**
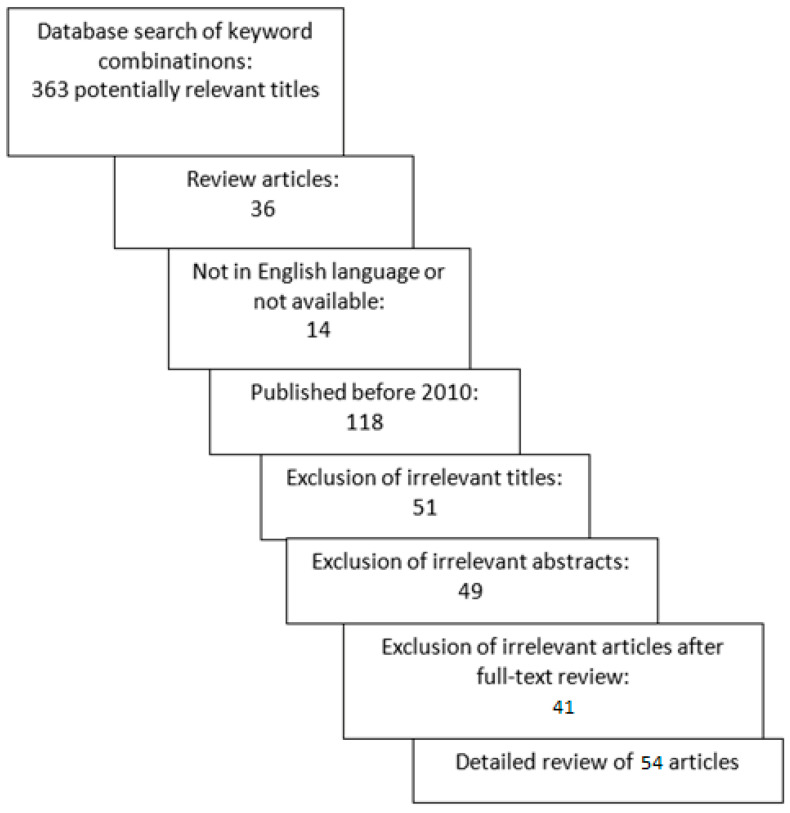
Search process for review of changes of extracellular matrix in cervical and endometrial cancer.

In our online database analysis, a total of 363 articles were identified. After excluding review articles, articles in other languages, and articles published before 2010, 195 articles remained for further review. After an initial review of the title and abstract, an additional 100 were excluded. We conducted a full-text review of the 95 articles, and ultimately, a total of 54 articles met the selection criteria and were included in the review. The selection process is shown in Figure 1.

## 3. Results

Proteins in the ECM are a focal component of the tumour microenvironment [19]. Significant changes in the basement membrane are observed during carcinogenesis. All of these proteins contribute to the specificity of the ECM [20]. A short overview of the main components described in our review is presented in Table 1.

### 3.1. Matrix Metalloproteinases

**Figure 2:**
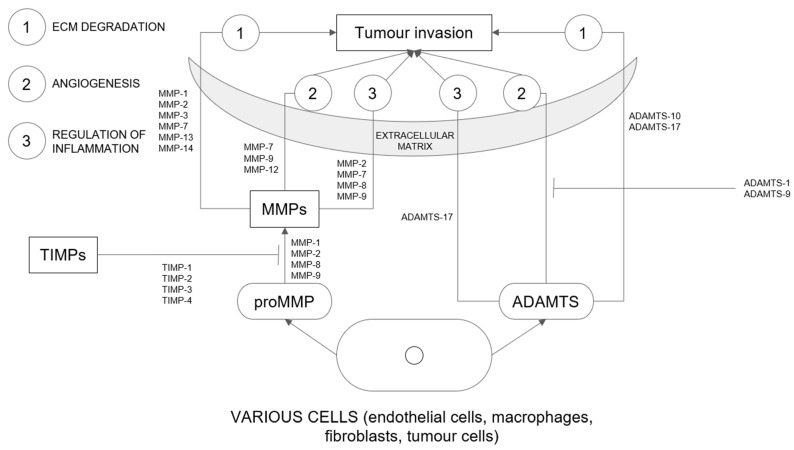
Main mechanisms of action of metalloproteinases, ADAMTSs and TIMPs in extracellular matrix. Abbreviations: ECM, extracellular matrix; MMP, metalloproteinase; TIMP, The tissue inhibitors of metalloproteinases; ADAMTS, A disintegrin and metalloproteinase with thrombospondin motifs.

Matrix metalloproteinases (MMPs) are a family of more than 20 enzymes [18,21]. Concerning their substrate specificity, they are divided into six subclasses (gelatinases, stromelysins, collagenases, membranous MMPs, matrilysins, and others) [18]. They play a key role in the degradation of extracellular matrix and basement membrane components (key mechanisms are presented in Figure 2) [21,22]. They not only contribute to physiological processes but are also responsible for molecular interactions between tumour and stroma [23]. Higher activity of MMPs has been associated with tumour invasion and metastasis in the course of destruction of extracellular matrix components [18,21,24,25]. On the other hand, inhibition of MMP activity has been shown to contribute to the reduction in cellular invasion in vitro and the progression of metastasis in vivo [26]. Increased expression and activation of MMPs are observed in most cancer tissues compared with normal tissues [18]. MMPs also contribute to cell proliferation, survival, and angiogenesis [21,24]. Several factors stimulate the synthesis, secretion, and activation of MMPs [21]. Physiologically, their expression is controlled by transcription [18]. MMPs, together with tissue inhibitors of metalloproteinases (TIMPs), play an important role in extracellular matrix transformation [24]. Tissue inhibitors of metalloproteinases (TIMPs) are inhibitors of disintegrin and metalloproteinase with thrombospondin motifs (ADAMTS) and MMPs. TIMPS, together with MMPs, are responsible for ECM transformation and are associated with tumour growth, invasion, and metastasis [27]. MMPs play an important role in cancer progression in both endometrial and cervical cancers. 

Gelatinase A (MMP-2) is an enzyme that degrades IV type collagen, a component of the basement membrane that is destroyed during cancer invasion. It can also use other types of collagens, elastin, and fibronectin as substrates. MMP-2 can also activate other matrix metalloproteinases [21]. In endometrial cancer, the overexpression of MMP-2 protein is related to the depth of myometrial invasion as well as histologic grade and FIGO (International Federation of Gynecology and Obstetrics) stage [21,25]. High expression of MMP-2 in combination with low expression of TIMP2 represents a high risk for local and distant metastasis of endometrial cancer. Both molecules could be an important marker for tumours [18]. MMP-2 plays also an important role in the degradation of ECM in cervical cancer and can also regulate other MMPs as well as components not found in the extracellular matrix, such as cytokines, chemokines, interleukins, and growth factors, adhesion, and growth factor receptors [28,29]. Shukla et al., comparing patients with cervical cancer to healthy individuals, found a positive correlation between increased serum levels of MMP-2 and higher cervical cancer stage. MMP-2 gradually increases with the increasing grade of cervical intraepithelial neoplasia and reaches a maximum in cervical cancer [28]. This was also confirmed by Azevedo Martins et al., where expression of proteins was obtained from biopsies of tumour and stroma. Increased expression of MMP-2 and TIMP-2 in the stroma were linked with lower survival. In reverse, the same study showed that increased levels of MMP-9 in tumour tissue, are associated with better survival compared to decreased levels [30]. 

Expression of MMP-11 in patients with endometrial cancer type 1 is also associated with poor prognosis. MMP-11 is the only MMP secreted in active form and is unable to degrade the major components of ECM [24]. It plays an important role in cancer progression and its overexpression could be used as a biomarker in endometrioid-type carcinomas. Gomez-Macias et al. pointed out that elevated MMP-11 levels could be associated with poor prognosis in patients with type I EC [24]. Skrzypczak et al. noted a slight change in MMP-11 expression levels in the G1 cancer subgroup, whereas there was no significant change in expression levels in the other subgroups, which may suggest that MMP-11 has a specific function in the early stages of carcinogenesis [30]. Endometrial cancer progression has also been associated with increased expression of MMP-2 and MMP-9 [18]. Poor prognosis in cervical cancer was also associated with higher expression of MMP-9 [31]. Previous studies have shown increased expression of MMP-2 and MMP-9 in pre- and postmenopausal patients with polyps, suggesting that their expression could depend on the hormonal status of patients with endometrial polyps and type I endometrial cancer. Both have negative prognostic value in the previously mentioned pathologies [3]. In cervical cancer, Schröpfer et al. confirmed the presence of MMP-2, -3, and -9. In their study, three cervical cancer cell lines, HeLa, Caski, and SiHa, were examined. High expression of MMP-14 mRNA was found in the Caski and SiHa cell lines, while MMP-1, -11, -13, -15, -17, -24, and -28 were expressed in all three cell lines. Analysis of these three cell lines could contribute to future studies of cervical cancer invasion [18].

### 3.2. A Disintegrin and Metalloproteinase with Thrombospondin Motifs (ADAMTS) 

A disintegrin and metalloproteinase with thrombospondin motifs (ADAMTS) are a family of 19 proteases [27,32]. A large number of ADAMTS are involved in different benign and malignant pathologies. ADAMTS metalloproteinases have four disulfide bonds that reinforce the structure, which distinguishes them from MMPs [27]. ADAMTS play a role in the degradation of the extracellular matrix and communicate with inflammatory cytokines (key mechanisms are presented in Figure 2). Through various processes, these proteases react with their substrates involved in ECM remodelling, angiogenesis, fibrosis, and coagulation [32]. These enzymes degrade ECM molecules such as collagen, versican, and aggrecan [27]. Increased activity of ADAMTS proteases contributes to various physiological and pathological processes [32]. 

ADAMTS 5 and ADAMTS 8 are well-studied genes in the process of chronic inflammation and tumorigenesis. ADAMTS 8 is a tumour suppressor and ADAMTS 5 contributes to tumorigenesis [32]. ADAMTS 1 and ADAMTS 9 also have tumour suppressive properties and antiangiogenic effects [27]. Yilmaz et al. found that serum levels of ADAMTS 5 were significantly higher in patients with endometrial cancer compared with patients with benign endometrial lesions. In the same study, serum levels of ADAMTS 8 were found to be decreased in patients with endometrial cancer compared with patients with benign endometrial pathologies [32]. ADAMTS 9 degrades aggrecan and versican in cancer cells, which are important components of its ECM. Serum levels of ADAMTS 9 were found to be significantly lower in patients with endometrial polyps compared to patients without pathological endometrial changes. Decreased ADAMTS 9 activity could be the reason for endometrial polyp formation via two possible mechanisms, either by preventing tumour ECM degradation or by enhancing angiogenesis of cancer cells. Its specificity is low, but it has a high sensitivity for endometrial polyps prediction. Tokmak et al. examined serum levels of ADAMTS 1 and ADAMTS 20 but found no statistically significant differences in women with endometrial polyps compared with women who had no pathological changes in the endometrium [27]. Qin Xu et al. showed higher expression of ADAMTS17 in cervical cancer tissues compared with normal cervical tissues and showed a correlation with poor prognosis [31].

**Table 1:** Short overview of MMPs and ADAMTSs and their action in tumour microenvironment.

Type of Cancer	Component of ECM	Action in Tumor Microenvironment
Endometrial cancer	MMP 2 [18,28,33,34]	Degradation of collagen type 4 in basal membrane
	MMP 9 [15,18,31,34]	Cancer progression, dependent on hormonal status
	MMP 11 [18,35]	Activation of other MMPs
	ADAMTS 1 [27]	Antiangiogenic effect
	ADAMTS 5 [32]	Involved in tumorigenesis
	ADAMTS 8 [32]	Tumour suppressor function
	ADAMTS 9 [27,32]	Antiangiogenic effect, degradation of aggrecan and versican
	ADAMTS 20 [27]	No statistically significant differences have been found between cancerous and healthy tissues
Cervical cancer	MMP 2 [28]	Degradation of ECM, regulation of other MMPs
	MMP 9 [15,34]	Associated with better survival rate
	MMP1, -3, -11, -13, -14, -15, -17, -24, -28 [18]	Found in Caski, HeLa and SiHa cell lines, their role in tumour development has not been fully understood yet
	ADAMTS17 [31]	Correlated with poor prognosis

### 3.3. MMPs and CD147

MMPs are interconnected with the extracellular matrix metalloproteinase inducer (EMMPRIN), also known as CD147. They belong to the immunoglobulin superfamily. CD147 is a transmembrane protein and is expressed on the surface of tumour cells. In endometrial cancer, these molecules promote the synthesis and secretion of MMPs in both mesenchymal cells and tumour cells [8,25]. CD147 secreted by endometrial tumour cells can stimulate the synthesis of CD147 by fibroblasts, which can lead to higher activation of MMPs in the stroma associated with the tumour [21]. Overexpression of CD147 in malignant cells contributes to invasion and metastasis and is linked to aggressive cancer and poor prognosis for patients. This suggests that CD147 plays an important role in tumour progression [8,25].

There is no significant difference in the expression of CD147 associated with the low or high FIGO stage of endometrioid endometrial carcinoma. Increased expression of CD147 has been shown to correlate with the expression of MMP-2. Yuan et al. showed that overexpression of CD147 was increased in patients with endometrioid endometrial carcinoma compared with patients with benign endometrial pathologies. There was a positive correlation between the expression of CD147 and MMP-2 and the clinicopathologic features of endometrioid endometrial cancer. Overexpression of CD147 is associated with lymph node metastasis, whereas overexpression of MMP-2 is associated with a higher FIGO stage, increased myometrial invasion, and high histologic grade. CD147 stimulates MMP-2 synthesis and promotes angiogenesis through the regulation of vascular endothelial growth factor (VEGF) expression. Elevated expression of CD147 and MMP-2 could be a reliable indicator of poor prognosis in endometrioid endometrial cancer [25]. In cervical cancer, the expression of CD147 is associated with the FIGO stage, lymph node metastasis, invasion of the parametrium, and differentiation. CD147 is involved in promoting tumour invasion and metastasis through stimulation of MMP production by stromal cells and local spread. Increased CD147 expression has been observed in squamous and adenocarcinoma tissues compared with normal cervical tissues [8,31]. Fan et al. indicated that overexpression of CD147 is significantly correlated with poor prognosis and its clinicopathologic features such as tumour stage, tumour differentiation, clinical stage, and nodal metastasis in different cancers, but not in cervical cancer [33]. 

### 3.4. Interplay of MMPs and Other Molecules 

An important consideration of MMPs in cervical cancer is also the interplay with the Human papillomavirus (HPV). It is associated with different clinical pathologies, from innocuous lesions to cancer. Chronic infection with high-risk HPVs is one of the main causes of cervical cancer [9,11,12]. HPV16, a high-risk HPV, is responsible for more than 50% of all cervical cancers [11,12]. E6 and E7, viral oncoproteins, interact with cellular host proteins, which leads to the transformation of the cells. Several studies have reported a correlation between HPV and increased expression of different MMPs. HPV16 infection is known to be a critical factor in the development of cervical cancer [13]. Kaewprag et al. proposed that HPV oncoproteins contribute to the upregulation of MMP2, which leads to the promotion of cervical cancer invasion. Their study showed that high-risk HPV16 infection contributes to increased invasion and thus poor prognosis of cervical cancer [11]. 

The receptor tyrosine kinase AXL is a member of the receptor family (tumour-associated macrophages) TAM and has been identified as a therapeutic target due to its role in tumour invasion and migration. Silencing of AXL is significantly associated with inhibition of expression of MMPs and uPA (urokinase-type plasminogen activator). This may be the pathway by which AXL mediates tumour metastasis. uPA plays an important role in ECM degradation and has been associated with a poor prognosis for EC patients [7].

Fibronectin has been shown to regulate MMP-2 and MMP-9 expression and activity in several cancers, including cervical cancer. Maity et al. indicated that SiHa cells on a fibronectin-coated surface correlate with activation of fibronectin–integrin-mediated signals leading to pro-MMP-2 and pro-MMP-9 expression and activation [29]. 

Laminin, basal lamina glycoprotein, has been shown to partake in the invasion and angiogenesis in different cancers, including cervical cancer. Its interaction with cancer cells is crucial for tumour invasion and metastasis. Laminin stimulates the expression of MMP-9 and its involvement in different cancers. It also stimulates the expression of MMP-2, therefore facilitating tumour invasion. Maity et al. reported that the SiHa cell line on a laminin-coated surface promoted the overexpression of MMP-9 and thus cell migration and invasion of the tumour [34]. Fullar et al. investigated the actions of normal and tumour fibroblasts in cervical cancer. Their study showed that their functions differ. Normal fibroblasts stimulate cell proliferation, whereas tumour fibroblasts promote the migration of cancer cells [36].

The plasminogen activator system (PA) is thought to be involved in the invasion and metastasis of cervical cancer, due to the link between plasmin regulation and extracellular matrix remodelling. The imbalance of this system can lead to numerous pathologies (thrombosis, type 2 diabetes), including cancer invasion and metastasis. Sato et al. examined the correlation between plasminogen activator inhibitor type 1 (PAI-1) and stem cells in cancer and noncancer cells. Their study suggests that due to low expression concentrations of PAI-1 in cancer stem cells, the ECM around them is more likely to be degraded [35].

Collagen plays an important role in stabilising the basement membrane and extracellular matrix of normal tissue. It is believed that type VIII collagen increases the synthesis of MMPs, which affects the migratory ability of cells. Significant overexpression of α-collagens of types VIII and XI has been found in stage III endometrial cancer tissue compared with normal endometrial tissue [20]. It is known that increased expression of collagen and integrin is related to matrix stiffness and thus to tumour development and progression. Overexpression of collagen-associated genes (COL4A4, COL19A1, COL6A6) occurs in the late stages of endometrial cancer, which could indicate that they partake in the progression of EC [6].

### 3.5. Tissue Inhibitors of Metalloproteinases (TIMPs)

TIMPs are inhibitors of ADAMTS and MMPs [27]. TIMPS, together with MMPs, are responsible for ECM transformation and have been associated with tumour growth, invasion, and metastasis. They play an important role in the modulation of different enzymes in the ECM [37,38]. Schröpfer et al. indicated that low expression of TIMP2 is a high risk for local and distant metastasis of endometrial cancer [18]. In cervical cancer studies show that in different cancers the roles of TIMPs are paradoxical, suggesting that they are tissue-specific. TIMP-4 is expressed de novo in cervical cancer cells and increases in advanced clinical stages. Lizarraga et al. discovered that its overexpression increases the sensitivity of HeLa cells to apoptosis through the modulation of apoptotic proteins [37,38]. Maity et al. proposed that the downregulation of TIMP1 and TIMP2 could be involved in higher activity of MMP2 and MMP9 [34]. Sidorkiewicz et.al. analysed plasma levels and tissue expression of metalloproteinases and tissue inhibitors in patients with cervical cancer, patients with high-grade cervical intraepithelial dysplasia (CIN3), and patients with ectropion. Higher concentrations of MMP9 and a lower concentration of TIMP2 were found in patients with cervical cancer and CIN3, compared to patients with ectropion. Increased MMP2 and MMP9 concentrations correlate with the stage of cervical cancer and the age of the patients. Deficient values of TIMP2 indicate that TIMPs are not able to inhibit greater amounts of MMPs, produced by cancerous cells, which leads to ECM degradation. In support of this conclusion, the study reported that overexpression of TIMPs reduced MMP activity, leading to inhibition of tumour environment remodelling [14].

### 3.6. Major ECM Adhesion Molecules

Our review identified proteoglycans (aggrecan and laminin), integrins (vitronectin), glycoproteins (nidogen) and fascin, an actin-bundling protein as major ECM components. 

Aggrecan and laminin belong to a group of proteoglycans. Aggrecan contributes to the adhesive and mitotic activity of cancer cells. Futyma et al. documented significantly increased expression of aggrecan in endometrial cancer cells in FIGO stage III classification compared with normal endometrial tissue. The fact that aggrecan was expressed only in grades 2 and 3 suggests that it may be a biomarker for cancer progression in a clinical setting [20]. Laminin is a major component of the basal lamina. Kashima et al. suggest that the laminin (gama)1 chain (encoded by the LAMC1 gene) is vital for promoting cell motility and endometrioid carcinoma invasion. In the in vitro study, Kashima et al. observed the LAMC1 expression on the entire surface of carcinoma cells. This suggests that overexpression of LAMC1 is linked to aggressive endometrioid endometrial cancer through proteoglycans [5].

Vitronectin is an integrin involved in different processes and is closely associated with cycle-dependent changes in endometrial cells. Increased expression is observed in the luteal phase of the menstrual cycle, but not in menopausal women or cancer. Increased vitronectin gene expression has been observed (compared with normal endometrial tissue), but only in stage III endometrial cancer, which may provide a link between vitronectin and the increased ability of cancer cells to metastasize and myometrial invasion [20]. 

Another molecule impacting adhesion in endometrial cancer was shown to be fascin, an actin-bundling protein that acts as a regulatory element in the maintenance and stability of actin. Fascin plays an important role in the regulation of cell adhesion, migration, and invasion. CD44v6, a transmembrane receptor protein of the adhesion molecule family, is expressed on the cell surface and plays an important role in ECM adhesion and other cellular processes. Both molecules are involved in ECM invasion. Gun et al. found a correlation between Fascin and histological grade. They also detected the expression of CD44v6, but without clinicopathologic correlation. They indicated that expression is a consequence of dysregulation and is not related to the progression of endometrial cancer [39]. 

In the basement membrane, nidogen has been shown to impact adhesion ability. Nidogen is a glycoprotein responsible for connecting the components of the basement membrane and controlling the proper interactions therein. It also plays an important role in cell adhesion ability, regulation of neutrophil chemotaxis and necrosis, and influences neoangiogenesis. Futyma et al. hypothesised that gene expression for this protein could be responsible for higher neoangiogenesis only in stage III endometrial carcinoma and prevents infiltration of the basement membrane by cancer cells [20].

### 3.7. Stromal ECM Components

Stroma signalling plays an important role in regulating the proliferative potential of the endometrial epithelium. Components of stroma partake in growth and cancer development [40]. The stromal components identified in this analysis were a proteoglycan (versican), the glycoprotein (SPARC) and the fibroblast-specific protein S100A4 [13].

Versican is a proteoglycan in the ECM. Its expression was predominantly observed in the stroma. Expression of versican is linked to lymph node metastasis, tumour size, infiltration depth, and vascular space involvement. The expression of versican does have an impact on survival in correlation with infiltration depth. In patients with cervical cancer with an infiltration depth greater than 15 mm, low expression of versican predicts poorer survival [41,42].

SPARC (secreted protein acidic and rich in cysteine) is a matricellular glycoprotein that modulates the interactions of cells with the surrounding ECM, thereby affecting underlying cell functions, namely, cell adhesion, proliferation, and differentiation. It is also involved in cancer progression via stromal cells. Yoshida et al. investigated its effect on stromal fibroblasts in endometrial cancer. The in vitro study indicated that overexpression of SPARC in endometrial cancer cells and fibronectin 1 jointly activate fibroblasts, promoting cancer progression and invasion [43].

The fibroblast-specific protein S100A4 promotes tumour growth by stimulating angiogenesis. It is secreted by tumour and stromal cells and is upregulated in adenocarcinomas and squamous cell carcinomas of the cervix. Tumour progression and consequently metastasis are strongly dependent on epithelial-mesenchymal transition (EMT). Several studies have shown that S100A4 expression correlates with EMT. Liu et al. investigated the interactions between S100A4 and E-cadherin in cervical cancer cells. They suggested that S100A4 could facilitate cervical cancer cell promotion and progression of cancer. E-cadherin, a transmembrane protein, mediates cell–cell and cell–extracellular matrix interactions. Previous studies have described its downregulation in cervical cancer. Decreased E-cadherin levels and overexpression of S100A4 have been associated with cervical cancer formation [13].

There are also significant changes in gene expression in the stromal cells surrounding endometrial cancer compared to healthy individuals. It was found that the expression of 605 genes altered in the stromal cells, with 330 genes downregulated and 275 upregulated. These genes are involved in various signalling pathways (Wnt signalling, cadherin signalling, ECM-receptor interaction, and focal adhesion). Of these 605 genes, 28 genes are involved in the Wnt signalling pathway. This pathway provides signal transduction from cell surface receptors into the cell. Activation of this pathway is closely linked to cancer. Many genes also belonged to the protocadherin family, a large family of cadherin-like cell adhesion proteins. Their adhesive characteristics play an important role in cell rearrangement, sorting, and migration [40]. Several studies have shown that lower cadherin expression is associated with extensive tumour invasion and metastasis in endometrial, ovarian, and cervical cancers [42].

### 3.8. Fibulins

Fibulins are a family of extracellular matrix proteins encoded by *FBLN* genes. They are responsible for the integrity and stability of basement membranes, elastic fibres, and loose connective tissue. It is a family of seven members involved in modulating cell morphology, growth, adhesion, and motility [16]. Fibulin-3, also known as EFEMP1 (epidermal growth factor-containing fibulin-like extracellular matrix protein 1) acts as a tumour suppressor in endometrial cancer. Yang et al. found that the concentration of fibulin-3 was decreased in EC compared to normal endometrium. Higher expression of fibulin-3 was observed in stage 1 endometrial cancer compared to stages 2 and 3. Decreased expression was also seen in patients with lymph node metastases. Fibulin-3 subdues the secretion of MMP-2 and MMP-9 [44]. The expression of fibulin-3 is downregulated by oestrogen [2]. Fibulin-4 is highly expressed in normal endometrial tissue. Low fibulin-4 expression is positively linked to the advanced cancer stage. High expression of fibulin-4 is also correlated with a better prognosis in patients with endometrial cancer [45,46]. Fibulin-5 is physiologically responsible for cell-to-cell and cell-to-matrix communication. Fibulin-5 has been found to be downregulated in endometrial cancer type 1 (at all FIGO stages). Winship et al. demonstrated that loss of fibulin-5 gene expression is associated with increased adhesion and proliferation of cells in vitro [47].

In cervical cancer, the expression of EFEMP1 is associated with neovascularization and poor prognosis in cervical cancer. Immunohistochemical studies have shown upregulation of fibulin-3 expression, which is linked to lymph node metastasis and vascular invasion, as well as more rapid tumour growth. Various studies have demonstrated different functions of fibulin-3 in promoting or inhibiting angiogenesis. The reason for such behaviour might be its interactions with other matrix proteins [16,48,49]. Poor prognosis and neovascularization are also associated with another member of the fibulin family, fibulin-4. In their study, Chen et al. found upregulation of fibulin-4 expression in cervical cancer and its connection with low differentiation, higher cancer stage, and positive lymph node status. Fibulin-4 was also increased in the serum of patients with carcinoma compared with healthy individuals. This could be helpful as a diagnostic and prognostic tool [46].

### 3.9. Cell Invasion

We identified ED13, CTHRC1, LOXL2 and COP9 as cell invasion molecules impacting tumour progression in the ECM of endometrial and cervical cancer. 

EDI3 (endometrial carcinoma differential 3) is a member of the glycerophoshodiesterase family of proteins and plays a key role in choline metabolism. EDI3 has been shown to play an important role in cellular migration regulation. Lesjak et al. showed a positive correlation between higher EDI3 expression and cell attachment, cell spreading, and integrin expression. Levels were higher in patients with endometrial cancer who later developed metastases [50].

CTHRC1 (Collagen triple helix repeat containing 1) is another ECM protein that is thought to play a significant role in cancer development. Lu-Ying et al. analysed its functional role in endometrial cancer progression and metastasis. Their study showed increased expression of CTHRC1 in EC tissue as opposed to normal endometrium. CTHRC1 was positively correlated with the expression of TAMs in EC, which is associated with poor prognosis. Therefore, TAMs can be used as therapeutic targets for the development of better outcomes in endometrial cancer [51]. CTHRC1 is also increased in cervical cancer and promotes cell migration and invasion in vitro and metastasis in vivo. It is secreted by cervical cancer cells and has a paracrine effect on regulating stromal cells in the microenvironment such as immune cells, fibroblasts, and endothelial cells. Zhang et al. demonstrated a correlation between E6/E7 oncoproteins and multiple ECM proteins regulation, particularly CTHRC1 [10].

Lysyl oxidase-like 2 (LOXL2), a member of the lysyl oxidase family, plays a critical role in the formation of the extracellular matrix by catalysing cross-links of elastin and collagen. It plays an important role in cancer cell proliferation, invasion, and metastasis. Cao et al. found that elevated values of LOXL2 correlate with a lower survival rate in cervical cancer [52]. 

The highly conserved constitutive photomorphogenesis 9 (COP9) signalosome (CSN) is associated with different types of cancers. CSN6, the most important subunit of CSN, is overexpressed in cervical cancer. Mao et al. showed a positive correlation of CSN6 with cervical cancer aggressiveness. Its upregulation promoted cancer cell migration and invasion, which are essential steps in cancer metastasis. This study suggests that CSN6 could be used as a prognostic marker and potential therapeutic target in cervical cancer [53].

## 4. Discussion 

In ECM, MMPs play an important role in both types of cancer. They degrade various specific substrates (Collagen, Elastin, Fibronectin, Aggrecan, Fibulin, Laminin, Tenascin, Vitronectin, Versican, Nidogen) and play a crucial role in the degradation of basement membrane and extracellular matrix components. Different studies suggest that their overexpression is associated with various pathologies, such as local invasion and metastasis. Similar types of MMPs are increased in both cancers, namely, MMP-1, MMP-2, MMP-9, and MMP-11. Previously mentioned studies have shown that the levels of MMP-2 and MMP-9 are significantly increased in both cancers compared with normal tissues and (or) benign lesions. Concentrations of MMP-2 and MMP-9 correlate with the FIGO stage and are associated with poor prognosis in endometrial cancer, whereas in cervical cancer, MMP-9 has been associated with a better outcome. MMP-11 was found to have a specific function in the early stages of carcinogenesis in endometrial cancer, as high levels were detected in early cancer stages and low or no detectable levels of MMP-11 were detected in later stages.

Our review shows ADAMTS were found elevated in cervical cancer tissues, particularly ADAMTS17, which was associated with a poorer prognosis. Elevated ADAMTS levels were also found in endometrial cancer, but their role is unclear. Yilmaz et al. found elevated ADAMTS5 levels in the serum of endometrial cancer patients. In the same study, decreased levels of ADAMTS8 were found. In another study, a statistically significant decrease in ADAMTS9 was found in patients with endometrial polyps compared with patients with endometrial cancer. Tokmak et al. detected increased serum levels of ADAMTS1 and ADAMTS20 in patients with endometrial polyps, but no significant difference from healthy patients. 

Tissue inhibitors of metalloproteinases are inhibitors of two classes of enzymes, MMPs and ADAMTS. Maity et al. suggested that downregulation of TIMP1 and TIMP2 could be involved in higher activity of MMP2 and MMP9 in both endometrial and cervical cancers. In support of this, Schröpfer et al. associated TIMP2 with an increased concentration of MMP2, suggesting that TIMP2 is with a higher risk of local and distant metastases. Lazarraga et al. suggested that TIMPs may be tissue-specific because of their paradoxical role in different cancers. In cervical cancer, TIMP4 was newly expressed and found to correlate with the advanced clinical stage. 

CD147 (EMMPRIN) is a transmembrane protein that plays an important role in the synthesis and secretion of MMPs in tumour cells, contributing to cancer invasion and metastasis. Previous studies have shown that CD147 positively correlates with the expression of MMP-2 and MMP-9 in both cancers, suggesting that patients with elevated concentrations of CD147 have a poorer prognosis. Laminin, a major component of the basal lamina, is suspected to promote cell motility and cancer invasion in various cancers. It stimulates the expression of MMP-2 and MMP-9 in endometrial and cervical cancers. Laminin is thought to be closely related to the aggressiveness of cancer, especially endometrioid cancer. CTHRC-1 promotes cell migration and invasion in both cancers. Its concentrations were greatly elevated in cancer cells as opposed to normal tissues. It is positively correlated with TAMs and therefore associated with poor prognosis.

Fibulins are responsible for the integrity and stability of basement membranes, elastic fibres, and loose connective tissue and are associated with neovascularization. Several studies have shown that fibulin-3 has different functions in promoting or inhibiting angiogenesis. It acts as a tumour suppressor in both types of cancer, as it can suppress the secretion of MMP-2 and MMP-9. Its levels have been found to be decreased in endometrial cancer. Higher expression of fibulin-3 was observed in stage 1 endometrial cancer compared to stages 2 and 3, suggesting that fibulin-3 expression negatively correlates with the cancer stage. Fibulin-4 is associated with low differentiation, a higher cancer stage, and positive lymph node status. Its levels were increased in the serum of patients with all stages of cervical cancer, while low levels were found in advanced-stage endometrial cancer. It has been suggested as a helpful diagnostic and prognostic factor.

### Roots of Further Exploration 

It is unclear what the impact of other molecules interconnected to the ECM such as IL11, FRAS1, OPN and EDIL3 is. These molecules represent further venues of exploration. Interleukin11 (IL11) is a member of the IL6 cytokine family and plays an important role in pregnancy and different pregnancy pathologies. In several studies, IL11 has been associated with trophoblast migration and invasion, the mechanisms of which are similar to those involved in cancer progression. Increased expression of IL11 has been found in endometrial cancer compared with normal endometrium, but its function in this cancer is still unknown. However, Lay et al. hypothesised that IL11 acts in endometrial cancer through the (p)-STAT3 pathway. Their study showed increased levels of IL11 in grade I tumours, which could be useful as a marker for EC in future studies [4]. Furthermore, FRAS1 as an extracellular matrix protein helps to regulate the adhesion of the epidermal-basement membrane and organogenesis in the developmental process. Mutations of the FRAS1 gene appear to cause Fraser syndrome. Recent studies associated its expression with cancer development but the mechanism of its production by cancer cells is still unknown. Jing Xu et al. expressed the possibility of using serum overexpression of FRAS1 as a prospective biomarker for endometrial cancer [54]. Osteopontin (OPN) is an important protein in the ECM that is involved in endometrial cancer progression through cell adhesion, angiogenesis, and metastasis. It is associated with type I collagen, fibronectin, and osteocalcin [22]. Al-Maghrabi et al. sought to determine whether there was a significant correlation between elevated osteopontin levels and clinicopathologic features of endometrial cancer in the in vitro study. They found higher osteopontin immunostaining in noncancerous endometrium compared with endometrial cancer. The same study showed that there was no statistically significant association between osteopontin immunostaining and tumour stage (FIGO stage) and tumour size, but there was evidence of better survival rates for patients found to have increased osteopontin immunostaining [1]. EDIL3 (Epidermal Growth Factor-like Repeats and Discoidin I-Like Domains 3) is an important ECM protein contributing to angiogenesis, and its expression is physiologically present only during embryonic development. Oplawski et al. investigated a correlation between EDIL3 expression and different grades of endometrial cancer. In the control group (patients without neoplastic changes), higher levels of EDIL3 occurred in glandular cells, whereas in the study group (patients with G1, G2, and G3 endometrial cancer), they occurred in cancer cells. The study showed a positive correlation between EDIL3 overexpression and cancer grade, with the greatest difference in expression occurring between G1 and G3 endometrial cancer. Overexpression of EDIL3 could distinguish normal endometrium from G1 endometrial cancer and could also be used as an adverse prognostic factor [19].

## 5. Conclusions

In conclusion, there are various changes in the extracellular matrix in endometrial and cervical cancer that lead to poor prognosis and could be used as potential biomarkers for invasion and metastasis. 

## Data Availability

Data is available upon reasonable request by the corresponding author.

## References

[1] Al-Maghrabi H., Gomaa W., Al-Maghrabi J. (2020). Increased Osteopontin Expression in Endometrial Carcinoma Is Associated with Better Survival Outcome. Ginekol. Pol..

[2] Yang T., Zhang H., Qiu H., Li B., Wang J., Du G., Ren C., Wan X. (2016). EFEMP1 Is Repressed by Estrogen and Inhibits the Epithelial-Mesenchymal Transition via Wnt/β-Catenin Signaling in Endometrial Carcinoma. Oncotarget.

[3] Peres G.F., Spadoto-Dias D., Bueloni-Dias F.N., Leite N.J., Elias L.V., Custódio Domingues M.A., Padovani C.R., Dias R. (2018). Immunohistochemical Expression of Hormone Receptors, Ki-67, Endoglin (CD105), Claudins 3 and 4, MMP-2 and-9 in Endometrial Polyps and Endometrial Cancer Type I. OncoTargets Ther..

[4] Lay V., Yap J., Sonderegger S., Dimitriadis E. (2012). Interleukin 11 Regulates Endometrial Cancer Cell Adhesion and Migration via STAT3. Int. J. Oncol..

[5] Kashima H., Wu R.C., Wang Y., Sinno A.K., Miyamoto T., Shiozawa T., Wang T.L., Fader A.N., Shih I.M. (2015). Laminin C1 Expression by Uterine Carcinoma Cells Is Associated with Tumor Progression. Gynecol. Oncol..

[6] Yadav V.K., Lee T.Y., Hsu J.B.K., Huang H.-D., Yang W.C.V., Chang T.H. (2020). Computational Analysis for Identification of the Extracellular Matrix Molecules Involved in Endometrial Cancer Progression. PLoS ONE.

[7] Divine L.M., Nguyen M.R., Meller E., Desai R.A., Arif B., Rankin E.B., Bligard K.H., Meyerson C., Hagemann I.S., Massad M. (2016). AXL Modulates Extracellular Matrix Protein Expression and Is Essential for Invasion and Metastasis in Endometrial Cancer. Oncotarget.

[8] Sato T., Watanabe M., Hashimoto K., Ota T., Akimoto N., Imada K., Nomizu M., Ito A. (2012). A Novel Functional Site of Extracellular Matrix Metalloproteinase Inducer (EMMPRIN) That Limits the Migration of Human Uterine Cervical Carcinoma Cells. Int. J. Oncol..

[9] Kong L., Wang J., Cheng J., Zang C., Chen F., Wang W., Zhao H., Wang Y., Wang D. (2020). Comprehensive Identification of the Human Secretome as Potential Indicators in Treatment Outcome of HPV-Positive and -Negative Cervical Cancer Patients. Gynecol. Obstet. Investig..

[10] Zhang R., Lu H., Lyu Y.Y., Yang X.M., Zhu L.Y., Yang G.D., Jiang P.C., Re Y., Song W.W., Wang J.H. (2017). E6/E7-P53-POU2F1-CTHRC1 Axis Promotes Cervical Cancer Metastasis and Activates Wnt/PCP Pathway. Sci. Rep..

[11] Kaewprag J., Umnajvijit W., Ngamkham J., Ponglikitmongkol M. (2013). HPV16 Oncoproteins Promote Cervical Cancer Invasiveness by Upregulating Specific Matrix Metalloproteinases. PLoS ONE.

[12] Balasubramaniam S.D., Wong K.K., Oon C.E., Balakrishnan V., Kaur G. (2020). Comparative Transcriptomic Profiling in HPV-Associated Cervical Carcinogenesis: Implication of MHC Class II and Immunoglobulin Heavy Chain Genes. Life Sci..

[13] Liu M., Liu J., Yang B., Gao X., Gao L.L., Kong Q.Y., Zhang P., Li H. (2017). Inversed Expression Patterns of S100A4 and E-Cadherin in Cervical Cancers: Implication in Epithelial–Mesenchymal Transition. Anatomical. Record.

[14] Sidorkiewicz I., Piskór B., Dąbrowska E., Guzińska-Ustymowicz K., Pryczynicz A., Zbucka-Krętowska M., Ławicki S. (2019). Plasma Levels and Tissue Expression of Selected Cytokines, Metalloproteinases and Tissue Inhibitors in Patients with Cervical Cancer. Anticancer Res..

[15] Azevedo Martins J.M., Rabelo-Santos S.H., do Amaral Westin M.C., Zeferino L.C. (2020). Tumoral and Stromal Expression of MMP-2, MMP-9, MMP-14, TIMP-1, TIMP-2, and VEGF-A in Cervical Cancer Patient Survival: A Competing Risk Analysis. BMC Cancer.

[16] Li J., Qi C., Liu X., Li C., Chen J., Shi M. (2018). Fibulin-3 Knockdown Inhibits Cervical Cancer Cell Growth and Metastasis in Vitro and in Vivo. Sci. Rep..

[17] Sahoo S.S., Yuan Quah M., Nielsen S., Atkins J., Au G.G., Cairns M.J., Nahar P., Lombard J.M., Tanwar P.S. (2017). Inhibition of Extracellular Matrix Mediated TGF-β Signalling Suppresses Endometrial Cancer Metastasis. Oncotarget.

[18] Schröpfer A., Kammerer U., Kapp M., Dietl J., Feix S., Anacker J. (2010). Expression Pattern of Matrix Metalloproteinases in Human Gynecological Cancer Cell Lines. BMC Cancer.

[19] Oplawski M., Dziobek K., Zmarzły N., Grabarek B., Tomala B., Leśniak E., Adwent I., Januszyk P., Dąbruś D., Boroń D. (2019). Evaluation of Changes in the Expression Pattern of EDIL3 in Different Grades of Endometrial Cancer. Curr. Pharm. Biotechnol..

[20] Futyma K., Miotła P., Rózyńska K., Zdunek M., Semczuk A., Rechberger T., Wojcierowski J. (2014). Expression of Genes Encoding Extracellular Matrix Proteins: A Macroarray Study. Oncol. Rep..

[21] Stewart C.J.R., Crook M.L. (2011). CD147 (EMMPRIN) and Matrix Metalloproteinase-2 Expression in Uterine Endometrioid Adenocarcinoma. Pathol. Res. Pract..

[22] Li Y., Xie Y., Cui D., Ma Y., Sui L., Zhu C., Kong H., Kong Y. (2015). Osteopontin Promotes Invasion, Migration and Epithelial-Mesenchymal Transition of Human Endometrial Carcinoma Cell HEC-1A through AKT and ERK1/2 Signaling. Cell. Physiol. Biochem..

[23] Baker T.M., Waheed S., Syed V. (2018). RNA Interference Screening Identifies Clathrin-B and Cofilin-1 as Mediators of MT1-MMP in Endometrial Cancer. Exp. Cell Res..

[24] Gómez-Macías G.S., Garza-Rodríguez M.L., Garza-Guajardo R., Monsiváis-Ovalle D., Ancer-Rodríguez J., Barrera-Saldaña H.A., Barboza-Quintana O. (2018). Overexpression of the Matrix Metalloproteinase 11 Gene Is a Potential Biomarker for Type 1 Endometrial Cancer. Oncol. Lett..

[25] Yuan Y., Shen N., Yang S.Y., Zhao L., Guan Y.M. (2015). Extracellular Matrix Metalloproteinase Inducer and Matrix Metalloproteinase-2 Overexpression Is Associated with Loss of Hormone Receptor Expression and Poor Prognosis in Endometrial Cancer. Oncol. Lett..

[26] Cho-Clark M., Larco D.O., Zahn B.R., Mani S.K., Wu T.J. (2015). GnRH-(1-5) Activates Matrix Metallopeptidase-9 to Release Epidermal Growth Factor and Promote Cellular Invasion. Mol. Cell Endocrinol..

[27] Tokmak A., Ozaksit G., Sarikaya E., Kuru-Pekcan M., Kosem A. (2019). Decreased ADAMTS-1, -9 and -20 Levels in Women with Endometrial Polyps: A Possible Link between Extracellular Matrix Proteases and Endometrial Pathologies*. J. Obstet. Gynaecol..

[28] Shukla S., Qureshi S., Singh U., Khattri S. (2020). A Study of Matrix Metalloproteinase-2 and Interleukin-18 in Preinvasive and Invasive Lesions of Cancer Cervix. J. Midlife Health.

[29] Maity G., Fahreen S., Banerji A., Roy Choudhury P., Sen T., Dutta A., Chatterjee A. (2010). Fibronectin-Integrin Mediated Signaling in Human Cervical Cancer Cells (SiHa). Mol. Cell. Biochem..

[30] Skrzypczak M., Springwald A., Lattrich C., Häring J., Schüler S., Ortmann O., Treeck O. (2012). Expression of Cysteine Protease Cathepsin L Is Increased in Endometrial Cancer and Correlates with Expression of Growth Regulatory Genes. Cancer Investig..

[31] Xu Q., Ying M., Chen G., Lin A., Xie Y., Ohara N., Zhou D. (2014). ADAM17 Is Associated with EMMPRIN and Predicts Poor Prognosis in Patients with Uterine Cervical Carcinoma. Tumor Biol..

[32] Yilmaz E., Melekoglu R., Taskapan C., Olmez Budak F., Toprak S. (2020). The Investigation of Serum Levels of ADAMTS 5 and 8 (the A Disintegrin and Metalloproteinase with Thrombospondin Motifs) in the Etiology of Endometrial Cancer. J. Obstet. Gynaecol..

[33] Fan H., Yi W., Wang C., Wang J. (2017). The Clinicopathological Significance and Prognostic Value of EMMPRIN Overexpression in Cancers: Evidence from 39 Cohort Studies. Oncotarget.

[34] Maity G., Sen T., Chatterjee A. (2011). Laminin Induces Matrix Metalloproteinase-9 Expression and Activation in Human Cervical Cancer Cell Line (SiHa). J. Cancer Res. Clin. Oncol..

[35] Sato M., Kawana K., Adachi K., Fujimoto A., Yoshida M., Nakamura H., Nishida H., Inoue T., Taguchi A., Takahashi J. (2016). Decreased Expression of the Plasminogen Activator Inhibitor Type 1 Is Involved in Degradation of Extracellular Matrix Surrounding Cervical Cancer Stem Cells. Int. J. Oncol..

[36] Fullár A., Dudás J., Oláh L., Hollósi P., Papp Z., Sobel G., Karászi K., Paku S., Baghy K., Kovalszky I. (2015). Remodeling of Extracellular Matrix by Normal and Tumor-Associated Fibroblasts Promotes Cervical Cancer Progression. BMC Cancer.

[37] Lizarraga F., Ceballos-Cancino G., Espinosa M., Vazquez-Santillan K., Maldonado V., Melendez-Zajgla J. (2015). Tissue Inhibitor of Metalloproteinase-4 Triggers Apoptosis in Cervical Cancer Cells. PLoS ONE.

[38] Lizarraga F., Espinosa M., Ceballos-Cancino G., Vazquez-Santillan K., Bahena-Ocampo I., Schwarz-Cruz y Celis A., Vega-Gordillo M., Garcia Lopez P., Maldonado V., Melendez-Zajgla J. (2016). Tissue Inhibitor of Metalloproteinases-4 (TIMP-4) Regulates Stemness in Cervical Cancer Cells. Mol. Carcinog..

[39] Dogan Gun B., Bahadir B., Bektas S., Barut F., Yurdakan G., Kandemir N.O., Oguz Ozdamar S. (2012). Clinicopathological Significance of Fascin and CD44v6 Expression in Endometrioid Carcinoma.

[40] Li M., Xin X.-Y., Jin Z.-S., Hua T., Wang H.-B., Wang H.-B. (2017). Transcriptomic Analysis of Stromal Cells from Patients with Endometrial Carcinoma. Int. J. Clin. Exp. Pathol..

[41] Gorter A., Zijlmans H.J., van Gent H., Trimbos J.B., Fleuren G.J., Jordanova E.S. (2010). Versican Expression Is Associated with Tumor-Infiltrating CD8-Positive T Cells and Infiltration Depth in Cervical Cancer. Mod. Pathol..

[42] Turan T., Torun M., Atalay F., Gönenç A. (2017). Assessment of Vitronectin, Soluble Epithelial-Cadherin and TGF-Β1 as a Serum Biomarker with Predictive Value for Endometrial and Ovarian Cancers. Turk. J. Pharm. Sci..

[43] Yoshida S., Asanoma K., Yagi H., Onoyama I., Hori E., Matsumura Y., Okugawa K., Yahata H., Kato K. (2021). Fibronectin Mediates Activation of Stromal Fibroblasts by SPARC in Endometrial Cancer Cells. BMC Cancer.

[44] Yang T., Qiu H., Bao W., Li B., Lu C., Du G., Luo X., Wang L., Wan X. (2013). Epigenetic Inactivation of EFEMP1 Is Associated with Tumor Suppressive Function in Endometrial Carcinoma. PLoS ONE.

[45] Wang T., Wang M., Fang S., Wang Q., Fang R., Chen J. (2017). Fibulin-4 Is Associated with Prognosis of Endometrial Cancer Patients and Inhibits Cancer Cell Invasion and Metastasis via Wnt/β-Catenin Signaling Pathway. Oncotarget.

[46] Chen J., Zhang J., Liu X., Fang R., Zhao Y., Ma D. (2014). Overexpression of Fibulin-4 Is Associated with Tumor Progression and Poor Prognosis in Patients with Cervical Carcinoma. Oncol. Rep..

[47] Winship A.L., Rainczuk K., Ton A., Dimitriadis E. (2016). Fibulin-5 Localisation in Human Endometrial Cancer Shifts from Epithelial to Stromal with Increasing Tumour Grade, and Silencing Promotes Endometrial Epithelial Cancer Cell Proliferation. Oncol. Lett..

[48] En-lin S., Sheng-guo C., Hua-qiao W. (2010). The Expression of EFEMP1 in Cervical Carcinoma and Its Relationship with Prognosis. Gynecol. Oncol..

[49] Song E.L., Hou Y.P., Yu S.P., Chen S.G., Huang J.T., Luo T., Kong L.P., Xu J., Wang H.Q. (2011). EFEMP1 Expression Promotes Angiogenesis and Accelerates the Growth of Cervical Cancer in Vivo. Gynecol. Oncol..

[50] Lesjak M.S., Marchan R., Stewart J.D., Rempel E., Rahnenführer J., Hengstler J.G. (2014). EDI3 Links Choline Metabolism to Integrin Expression, Cell Adhesion and Spreading. Cell Adh. Migr..

[51] Li L.Y., Yin K.M., Bai Y.H., Zhang Z.G., Di W., Zhang S. (2019). CTHRC1 Promotes M2-like Macrophage Recruitment and Myometrial Invasion in Endometrial Carcinoma by Integrin-Akt Signaling Pathway. Clin. Exp. Metastasis.

[52] Cao C., Lin S., Zhi W., Lazare C., Meng Y., Wu P., Gao P., Wei J., Wu P. (2020). LOXL2 Expression Status Is Correlated With Molecular Characterizations of Cervical Carcinoma and Associated With Poor Cancer Survival via Epithelial-Mesenchymal Transition (EMT) Phenotype. Front. Oncol..

[53] Mao Z., Sang M.M., Chen C., Zhu W.T., Gong Y.-S., Pei D.S. (2019). CSN6 Promotes the Migration and Invasion of Cervical Cancer Cells by Inhibiting Autophagic Degradation of Cathepsin L.. Int. J. Biol. Sci..

[54] Xu J., Min W., Liu X., Xie C., Tang J., Yi T., Li Z., Zhao X. (2012). Identification of FRAS1 as a Human Endometrial Carcinoma-Derived Protein in Serum of Xenograft Model. Gynecol. Oncol..

